# Physical activity levels in female breast cancer patients and survivors in Ekurhuleni, South Africa

**DOI:** 10.17159/2078-516X/2023/v35i1a16001

**Published:** 2023-10-05

**Authors:** R Wilkinson, L Smith

**Affiliations:** Department of Sport and Movement Studies in the Faculty of Health Sciences, University of Johannesburg, Doornfontein Campus, Johannesburg, South Africa

**Keywords:** exercise, rehabilitation, oncology and municipal

## Abstract

**Background:**

By using complementary therapies, such as exercise rehabilitation during and after cancer treatment, breast cancer patients and survivors can improve their quality of life and overall health while also negating the deleterious effects of breast cancer and its treatment.

**Objectives:**

The aim of this study was to determine the physical activity levels of female breast cancer patients and survivors in Ekurhuleni, South Africa.

**Methods:**

The International Global Physical Activity Questionnaire (2002) determined participants’ physical activity levels during work, travel and leisure. The questionnaire was disseminated to medical facilities in hard copy format and online via the Google Forms platform. Statistics were computed using the Statistical Package for Social Science (SPSS) with the level of significance set at 95% (p < 0.05).

**Results:**

One hundred female breast cancer patients and survivors with a mean age of 55 years from Ekurhuleni, South Africa participated in this study. The findings reflected that most participants (59%) were meeting the American College of Sports Medicine’s physical activity guidelines when considering activity done during work, travel and leisure. No significant difference was seen in physical activity participation between breast cancer patients and breast cancer survivors, or those attending private and public facilities.

**Conclusion:**

For the breast cancer patient, physical activity and exercise may be a promising and effective adjuvant treatment both during and after anticancer therapies, improving quality of life, playing a role in increasing treatment tolerance, mitigating a range of symptoms and side effects brought on by cancer diagnosis and treatments and enhancing outcomes.

In South Africa, recent cancer statistics from 2019 show that, nationally, there were 10 172 new breast cancer diagnoses among females and 184 among males. ^[1]^ The most recent municipal cancer data for South Africa was collected in 2018 in Ekurhuleni, Gauteng, which saw breast cancer contributing to 12% of all cancer cases in the region, with 553 females diagnosed with this disease. ^[2]^

As an adjuvant therapy, exercise is becoming increasingly important in the management of cancer patients and survivors. ^[3]^ Research has demonstrated that exercise is both safe and possible for breast cancer patients and survivors, offering a range of physical and psychological benefits. ^[3,4]^ Such benefits include improvements in physical functioning, improved treatment outcomes and quality of life, reducing both the risk of developing breast cancer and the risk of its recurrence, as well as lowering the relative risk of all-cause mortality. ^[3, 4]^ Research has shown that in females with breast cancer, physical activity levels on average decreases by two hours per week from prediagnosis to postdiagnosis. ^[5]^

When encouraging physical activity and exercise participation in this population, it is crucial to remember some of the possible causes of the decline in physical activity: breast cancer, its treatments, and age-related decline. ^[5]^ While much research is still needed on patterns of physical activity and optimal physical activity and exercise implementation for breast cancer patients and survivors, evidence demonstrates that being physically active is safe and contributes to these patients’ physical and psychosocial health. ^[4, 5, 6]^ Physical activity can provide a cost-effective way to prevent and manage the burden of breast cancer, while being safe for this condition, and benefiting quality of life. ^[5, 6]^ The aim of this study was to determine the physical activity levels of female breast cancer patients and survivors in Ekurhuleni, South Africa.

## Methods

### Study design

The study was cross-sectional in design, making use of quantitative data. The use of this study design enabled the researchers to collect observational data, by measuring physical activity levels amongst females who had been diagnosed with breast cancer. The quantitative nature of the data allowed for descriptive and comparative analyses to be completed.

### Selection and recruitment

All participants who met the inclusion criteria of this study were invited to participate by completing the questionnaire, either online, through the Google Forms platform, or a hard copy. Recruitment occurred by making the link and hard copies available for qualifying participants at various private and public healthcare facilities and practices, and making the link available online on social media platforms and breast cancer support groups. A total of 100 females participated in the study in 2021 and 2022. The majority of the participation (89%) was completed online, through the Google Forms platform.

### Inclusion criteria

- Female participants who had been clinically diagnosed with breast cancer.
- Patients undergoing treatment (chemotherapy, radiation therapy, surgery, hormonal therapy, etc.) for breast cancer and those who had survived breast cancer.
- Participants residing in the Ekurhuleni municipality of Gauteng, South Africa at the time of data collection.
- Access to the internet for completion of the online questionnaire.

### Ethical considerations

Before the start of this study, ethical clearance was granted from the Research Ethics Committee of the Faculty of Health Sciences at the University of Johannesburg. Permission was sought and granted from relevant health care facilities and their practitioners before distribution of the study, as well as permission from the National Health Research Database. All participants were informed of the purpose of the study through an information letter preceding both the online and hard-copy versions of the questionnaire and were required to provide consent before commencing participation.

### Questionnaires

The first set of questions was related to basic demographics and breast cancer diagnosis of the participant for statistical purposes. Although this section obtained demographics, no personal information was gathered, therefore making participation anonymous. The rest of the questionnaire made use of the Global Physical Activity Questionnaire (GPAQ) which uses quantitative questions to describe physical activity participation during work, travel, and leisure-based activities. The GPAQ is widely used and considered a valid and reliable questionnaire for healthy adults.

### Statistical analysis

Statistics for this study were descriptive and inferential. Statistical analysis was completed using the Statistical Package for Social Sciences (SPSS, Version 28), which included descriptives and associations. Descriptive data analysis included percentages, means and standard deviations. Cross-tabulations were computed using Fisher’s exact test to assess for significant associations in physical activity levels between breast cancer patients and survivors, and the type of healthcare facility attended for care.

## Results

### Demographics

The current mean age of the participants was 55 years, with a mean age of breast cancer diagnosis of 50 years. Forty-two per cent of the participants were undergoing treatment at the time of their participation.[Table t1-2078-516x-35-v35i1a16001][Table t2-2078-516x-35-v35i1a16001]

Regarding remission status, 67% of the participants reported they were currently in remission for their breast cancer and were classified as breast cancer survivors for further analysis. One per cent reported that they were not in remission but were no longer receiving treatment. Of those who were in remission, the reported period of remission was 14%, 38% and 46% for less than one year, 1–5 years and more than 5 years, respectively. In this study, 79% of the participants reported that they attended private healthcare facilities for the diagnosis and treatment of their breast cancer.

Participants were asked to indicate the stage of breast cancer with which they were diagnosed. The categories were Stage I (25%), Stage II (35%), Stage III (17%) and Stage IV (7%) respectively. Sixteen percent of the participants selected stated that they did not know the stage of their breast cancer diagnosis.

The most commonly reported treatment modality was surgery (82%), with chemotherapy (60%) being the second most reported treatment. The other treatment modalities specified were electromagnetic field therapy; Femara, Herceptin and Denosumab; holistic; Lucrin; Tamoflex; Tamoplex; Tamoxifen; and targeted gene therapy.

### Physical activity

Of the 100 participants, 29% were unemployed, therefore they did not complete the questions regarding physical activity at work. With regard to the intensity of activity, both vigorous (5% of participants) and moderate (34% of participants) was completed during work, with both intensities having a mean of three days. However, moderate intensity activity had a greater mean amount of time by seven minutes per day. Thirty-four percent of participants indicated that they walk or cycle for travel purposes for a mean of 4.5 days per week for a mean amount of time of 48 minutes. Twenty-five and thirty-six percent of participants partook in vigorous and moderate intensity physical activity respectively during leisure time, both with a mean of three days per week and a similar mean amount of time in minutes (59 and 57 minutes respectively).

Further analysis was completed to classify the participants as physically active or physically inactive according to the ACSM’s Guidelines for Physical Activity. ^[7]^ Of the participants in this study, considering all three modalities of activity, 59% met the ACSM’s guidelines and were therefore classified as physically active. If considering only leisure activity, 32% of participants met the requirements.

Twenty-five percent of the participants reported not engaging in any form of physical activity. The leisure category had the highest number of participants, with 19%. The work and travel category had the least number of participants, at 5%. Eight percent of the participants participated in physical activity in all three modalities.

To quantify sedentary time, participants were required to select the time spent seated or reclining on a typical day. The options of choice were 1 to 2 hours (26%), 3 to 4 hours (30%), 5 to 6 hours (26%), and more than 7 hours (18%).

### Associations

Table 3 shows physical activity classification between those attending private facilities compared to those attending public facilities and between breast cancer patients and breast cancer survivors.

Of those who attended private facilities for their breast cancer diagnosis and treatment, 58% were meeting the physical activity guidelines, with a slightly higher percentage seen in those attending public facilities (62%). Fisher’s exact test was computed to assess for an association between the two categorical variables. Because the score was 0.808, no statistically significant difference was seen between physical activity classification and the type of facility attended. In the breast cancer survivor population, 61% of the participants were meeting the physical activity guidelines and were therefore classified as physically active. A smaller percentage, namely, 55% of breast cancer patients, were classed as physically active. Fisher’s exact test was completed to assess for an association between the two categorical variables. The result of 0.666 meant that no statistically significant difference was noted between physical activity classification and the patient or survivor status of the participant.[Fig f1-2078-516x-35-v35i1a16001]

## Discussion

### Demographics

The study recruited breast cancer patients and survivors residing in the Ekurhuleni region of Gauteng, South Africa. This municipal region accounts for some of the more recent area-specific cancer statistics in the country, which showed that 15 men and 553 women in the region were diagnosed with breast cancer in 2018. ^[2]^ There is a lack of an updated cancer registry for South Africa, making it difficult to understand the exact prevalence and mortality of breast cancer in the country and in specific regions. ^[8]^

There are various treatment modalities for breast cancer, and the modality is chosen based on location, type, stage and grade of the cancer, as well as the patient’s general health status. ^[9]^ Surgery was the most reported treatment modality (82%) in this study, which is consistent with research by Lambert et al. ^[10]^ Although adjuvant therapy has been proven to enhance breast cancer outcomes in terms of reduced reoccurrence and mortality, it commonly gives rise to unfavourable side effects. ^[11]^ Chemotherapy and radiation therapy were the second and third most reported modalities of treatment received (60% and 44%, respectively) by participants in this study.

### Physical activity

Physical activity is encouraged by the WHO as a tool to combat the increased chronic diseases and illness mortality risks in general and has attracted attention in the rehabilitation of cancer patients. ^[4]^ Research has shown a statistically significant inverse association between exercise participation and breast cancer mortality as well as between exercise and breast cancer reoccurrence. ^[3]^ The type of breast cancer, treatment modalities, and physical qualities before cancer may affect an individual’s ability to be physically active during and after breast cancer diagnosis and treatment. ^[12]^

The general exercise recommendation for breast cancer survivors, which is similar to that of the general population, is to perform 150 minutes of moderate or 75 minutes of vigorous intensity exercise per week. ^[7]^ Generally, breast cancer patients often experience a decline in their physical activity levels during and after their treatments. ^[6, 12, 13]^ Further research has shown that physical activity levels in women with breast cancer, on average, decreases by two hours per week from the pre-diagnosis to post-diagnosis phase. ^[5]^ This decline in physical activity may be associated with the combined effect of breast cancer diagnosis and treatment. ^[11]^ However, contrary to research and the hypothesis of this study, the majority of participants (59%) in this study were classified as physically active. This classification relied upon the American College of Sports Medicine’s Guidelines for Exercise Testing and Prescription of 150 minutes of moderate and/or 75 minutes of vigorous intensity activity per week. ^[7]^ The result is promising to this population’s well-being due to the numerous associated health benefits of being physically active, with exercise proving to be an important adjunct therapy for the breast cancer population. ^[3, 5, 9,]^

In this study, 25% of participants reported participating in no form of physical activity and were therefore classed as sedentary, while on the other end of the scale 8% participated in physical activity during work, travel and leisure. Sixteen percent of participants in this study were classed as neither sedentary nor physically active as, although they were participating in physical activity, they did not meet the required guidelines.

Exercise is recommended as part of the cancer care plan, with these patients being ‘as physically active as their conditions and abilities allow’. ^[6, 14]^ One also needs to consider the disability and morbidity caused by breast cancer and its treatment, which can negatively affect physical activity levels and consequently impact quality of life. ^[6, 14]^ In Avancini et al., a multivariate logistic regression showed that willingness to participate in exercise was associated with two main factors: education and current physical activity participation. ^[15]^

When considering only leisure activity, 32% of participants were meeting the guidelines to be classed as physically active. If one is classed as inactive or active, it is important to consider not only their exercise levels (leisure), as it is possible to be physically active in three other categories: work, household, and transport. ^[5]^ Interestingly, the four categories of physical activity (leisure, household, occupational and travel) have each been shown to prevent breast cancer with a 21%, 21%, 18% and 13% reduction in risk, respectively. ^[5]^ This study demonstrates the difference in physical activity classification when only considering leisure-based activity (32%) and when considering all modalities of physical activity (59%), which may not always be considered when looking at physical activity prevalence studies in general. Nevertheless, the benefits of being physically active are independent of whether the activity is done for recreation, work, travel or in the household. ^[4]^ That being said, cancer patients and survivors are encouraged to complete aerobic, resistance, flexibility, breathing and balance modalities of exercise respectively as each carries its own benefits and together are considered as a well-rounded exercise programme. ^[16]^ One of great advantages of exercise is that there are various modes and intensities that one can do according to one’s preferences and abilities, allowing exercise to be attainable for all individuals. ^[17]^

In this study, 34% and 6% of participants reported completing moderate and vigorous intensity activity respectively, with 22% of participants participating in both moderate and vigorous intensity activities. Research has shown that both moderate (15%) and vigorous (18%) intensity exercise can reduce breast cancer risk. ^[5]^ Moderate intensity activity is regarded as safe during and after cancer treatment, with the benefits far outweighing the risks. ^[5]^ Lower intensity exercise is often advised for those who are deconditioned and still initiated in exercise participation; however, more vigorous intensity physical activity is seen to have added benefits in terms of reducing cancer mortality compared to moderate intensities. ^[4]^

Physical activity conversations should occur between physicians and cancer patients, as this may encourage and aid the cancer patients to take up physical activity. ^[18]^ Indeed, it is important for both breast cancer patients and survivors to receive clearance from their doctors before partaking in exercise. ^[17, 18]^ Exercise for breast cancer patients and survivors carries similar and a few additional risks to that of the general population, which should always be considered. Patients should be educated on these associated risks and how to exercise safely. ^[13]^

When assessing one’s activity levels, it is also essential to consider sedentary time. Notably, inactivity and being sedentary are not the same and do not carry the same health effects. ^[6]^ Sedentary activities include those requiring less than 1.5 METs, and include behaviours such as sitting, reclining or lying down. ^[19]^ Breast cancer patients and survivors risk becoming sedentary for numerous reasons, and all-cause mortality is adversely associated with sedentary behaviour. ^[4, 6, 19]^ In this study, 25% reported participating in no forms of physical activity. Small physical activity adjustments through activities of daily living, such as using the stairs instead of an elevator, are ways that these patients and survivors can become more physically active and start an active, healthy lifestyle. ^[4]^ In some patients, sitting and standing up and walking around their home contributes sufficiently to physical activity in order to see some of these benefits. ^[17]^

The final question of the GPAQ elicits data about time spent sedentary on a typical day. In this study, 26% spent 1–2 hours seated, 30% 3–4 hours, 26% 5–6 hours and 18% more than 7 hours seated. In a study by van der Ploeg et al., all-cause mortality hazard regression analysis was 1.02 for 4–8 hours of sitting time, 1.15 for 8–11 hours and 1.40 for more than 11 hours compared to those sitting for less than four hours per day. ^[19]^ These results suggest an increase of 11% all-cause mortality risk for a rise in sitting time. ^[19]^ Protection from all-cause mortality can be seen by meeting the physical activity guidelines, as well as sitting for less than eight hours per day. ^[19]^

Research has shown the value of exercise in the breast cancer survivors’ trajectory. ^[5]^ Physical activity may assist breast cancer patients and survivors to cope and recover from their treatment, improve longevity, and possibly reduce the risk of cancer reoccurrence. ^[4]^ In Campbell et al. ^[9]^ specific exercise guidelines have been provided for breast cancer survivors. However, further research is needed on the optimal modality, intensity, frequency and volume of exercise for breast cancer patients receiving treatment and patients with metastatic breast cancer. ^[3, 11]^ Exercise is unquestionably beneficial for general health; however, physicians, relatives, and breast cancer patients themselves are often hesitant in allowing the latter to participate in exercise during cancer treatments and are often uncertain of what type, intensity and volume of exercise may be the most effective and safest. ^[4]^

### Differences in physical activity levels exist between types of facilities attended and breast cancer status

#### Public and private facilities

In South Africa, medical care can either be sought through public facilities, for which there is no charge for South African citizens, or private medical care through medical aid schemes, or by paying directly out-of-pocket. ^[20]^ In 2017, 17% of South African adults were privately insured for medical care. ^[20]^ Most participants (79%) in this study attended a private facility for their breast cancer diagnosis and treatments, which the researchers acknowledge is a limitation of the study in terms of comparative data.

In terms of the type of facility where participants were receiving care, it was hypothesised that those attending private facilities would be more physically active as they may be in a better financial position, and therefore possibly have greater access to leisure-based activity due to the associated cost of gym memberships, sports clubs and equipment. However, in contrast to the researcher’s null hypothesis, this study saw a slightly greater percentage of public facility patients meeting the physical activity guidelines. Including work and travel activities in the physical activity classification may have resulted in this unexpected result, and may potentially relate to the fact that those attending public facilities are in a less well-off financial position. Also, they may not have access to personal transport, therefore requiring more activity to get between places. Moreover, their work may be more physically demanding and laborious. These reasons may offer justifications for the result seen in this study, where the percentages of those physically active were not significantly different among those attending private (58%) and those attending public (62%) healthcare facilities.

#### Breast cancer status

Individuals may be affected by breast cancer to different degrees, and it is recommended that exercise be part of the cancer continuum of care, from diagnosis to treatment and recovery. ^[14]^ In terms of the differences in activity levels between breast cancer patients and breast cancer survivors, as hypothesised by the researchers, the breast cancer survivor population had a greater percentage of participants reaching the physical activity guidelines (61% compared to 55%). This is somewhat to be expected, as those currently undergoing treatment may be dealing with acute side effects which a survivor may no longer be facing. ^[5]^ Fairness regarding the use of physical activity classification may be in question for breast cancer patients who are currently undergoing treatment, as these are the guidelines for breast cancer survivors. However, no guidelines for physical activity classification for the in-treatment breast cancer population exist.

### Study limitations

The sample size of this study is one of the main limitations. The researcher made the questionnaire available at both public and private facilities, as well as on an online platform. However, hesitancy in hard-copy participation was evident and some may have wanted to participate online but did not have the means to do so.

The limitations of this study include:

- Only female breast cancer patients and survivors and those residing in the Ekurhuleni region of South Africa could participate in the study. These criteria excluded the male population and those in surrounding areas, as previously mentioned. This was done in order for the present study to be specific.
- Due to the online nature of the questionnaire, there may have been numerous breast cancer patients and survivors who were unable to participate in the research, due to a lack of resources, such as internet access. The link was used to create a QR-code which allowed for quick access to the questionnaire; however, the elderly and technologically challenged participants may not have understood how to access the QR-code. Physical hard-copies of the questionnaire were offered to numerous hospitals and medical facilities; however, due to the risk of COVID-19 some opted not to distribute hard-copies to their patients and some patients were not comfortable completing a hard-copy questionnaire.
- The questionnaire was in English. This was due to the associated cost of translating and distributing the questionnaire in the other South African languages, thereby limiting non-English literate members of the population.
- The questionnaire did not investigate the type of exercise these participants were partaking in, which could have added further value to the study.
- The difference in responses with regards to exercise habits could have been affected by variable factors such as a current treatment phase, the COVID-19 pandemic, stage of disease, prescribed medications and/or daily experience around the time of participation.
- Fairness regarding the use of physical activity classification may be in question, for breast cancer patients who are currently undergoing treatment. However, no guidelines for physical activity classification for this population exist. The researchers acknowledges the limitations breast cancer treatment may place on this population’s ability to meet the guidelines set out for breast cancer survivors who have completed treatment.
- Validity and reliability of the GPAQ in this population has not yet been determined and therefore the researchers acknowledge that other instruments may have been more appropriate for this study.
- The researchers acknowledge that the use of self-reported activity questionnaires may result in an overestimation of activity levels, compared to collecting direct observations.
- Finally, physically active breast cancer patients and survivors may have been more inclined to participate than their less active counterparts, which may have led to selection bias.

### Recommendations for future research

As cancer research is continuing, as well as there being an increase in the cancer survival rate, further research is required on the holistic approach to cancer treatment and recovery to allow for optimal results in terms of life expectancy and quality of life. ^[3]^ Research on the holistic care of the cancer patient and survivor is needed, to allow the potential for them to achieve an optimal quality of life both during and after their cancer journey. This research opens up inspiration for similar studies to be conducted in other regions of South Africa as well as other countries. Research on exercise attitudes, knowledge and preferences among the population will provide further insights in being able to encourage and prescribe activity participation. Further research and implementation in terms of health education in both breast cancer and clinician populations on the importance and application of physical activity is needed. This study opens further research questions regarding the optimal physical activity prescription for the breast cancer population as a whole, and in terms of specific cancer stages, types, and treatments.

## Conclusion

Advances in understanding breast cancer have seen progress in breast cancer care as well as more holistic approaches to management. ^[5, 11]^ The role of physical activity is becoming increasingly important for cancer prevention, optimising recovery, limiting deterioration, controlling associated symptoms and improving chances of survival. ^[6]^ The field of oncology can greatly benefit from the inclusion of physical activity as part of cancer care, as it offers many benefits in terms of primary prevention, as well as coping and recovering from treatments, improving long-term health and reducing risk of cancer reoccurrence. ^[4]^ This study provided preliminary information regarding physical activity participation in a South African breast cancer population.

## Figures and Tables

**Fig. 1 f1-2078-516x-35-v35i1a16001:**
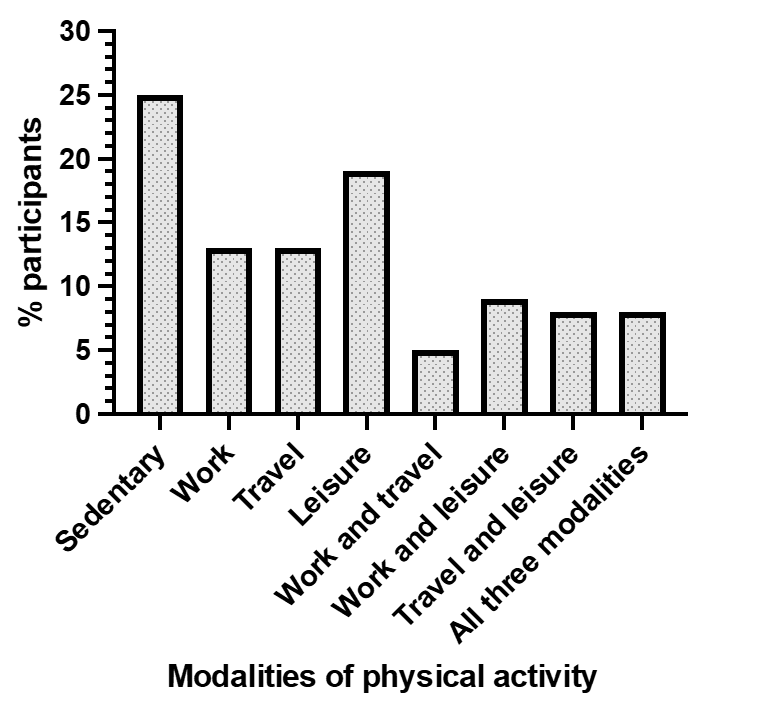
Modalities of physical activity completed by the participants (n=100)

**Table 1 t1-2078-516x-35-v35i1a16001:** Demographic data of the sample (n=100)

Variable	Response	Percentage (%)

**Current age (years)**	20–29	1
	30–39	8
	40–49	18
	50–59	37
	60–69	25
	70–79	8
	80–89	3

**Age at breast cancer diagnosis (years)**	20–29	1
	30–39	15
	40–49	34
	50–59	31
	60–69	15
	70–79	3
	80–89	1

**Currently undergoing treatment**	Yes	42
	No	58

**Type of treatment received** *	Surgery / Operation	82
	Chemotherapy	60
	Radiation therapy	44
	Hormone therapy	38
	Decided not to have treatment	2
	Other (specified)	13

* Participants could select multiple responses

**Table 2 t2-2078-516x-35-v35i1a16001:** Global Physical Activity questionnaire results (n=100)

Activity	Response	Percentage (%)	Number of days	Duration (Minutes)
**Vigorous intensity activity during work** *	Yes	5	3.8 ± 1.3	126 ± 105
	No	66		
**Moderate intensity activity during work** *	Yes	34	3.8 ± 1.4	133 ± 106
	No	37		
**Activity during travel**	Yes	34	4.5 ± 1.7	48 ± 38
	No	66		
**Vigorous intensity activity during leisure**	Yes	25	3.2 ± 1.2	59 ± 35
	No	75		
**Moderate intensity activity during leisure**	Yes	36	3.1 ± 1.2	57 ± 53
	No	64		

Data are expressed as percentage or as mean ± SD.

* 29% of participants are unemployed

**Table 3:** Physical activity classification by facility and breast cancer status (n=100)

	Physically active (%)	Physically inactive (%)	Fisher’s exact test

**Type of facility**			
Private facility	58	42	0.808
Public facility	62	38	

**Breast cancer status**			
Breast cancer survivor	61	39	0.666
Breast cancer patients	55	45	
